# Supplementary material for: A qualitative analysis of the impact of COVID-19 restrictions on gender biases in an Irish University
Source: PLoS One. 2023 Sep 27;18(9):e0288467. doi: 10.1371/journal.pone.0288467 (PMC10529590; doi:10.1371/journal.pone.0288467)
Supplement: S1 File — (DOCX) [file pone.0288467.s001.docx]

# S1. Supplemental Document

Information sheet…………………..…………………..…………………..…………………. 2

Informed consent form…………………..…………………..…………………..…………… 4

Questionnaire…………………..…………………..…………………..…………………..… 6

COREQ Checklist…………………..…………………..…………………..……………….. 8

Results of the Likert-Type Questionnaire…………………..…………………..…………… 12

# Information sheet

**Title of the research**: the impact of COVID-19 restrictions on gender bias in academia

This research aims to explore the impact of COVID-19 restrictions on gender bias in academia via interviewing members of a STEM Faculty in Ireland. In this research, gender bias is understood as gender-based inclinations or prejudices that affect researchers’ personal and professional opportunities. Investigators are Mohammad Hosseini and Alicia Castillo Villanueva, and the interviews will be conducted by either one or both investigators.

This project has received funding from [UNIVERSITY] Faculty of [FACULTY NAME].

**Why is the research being conducted?**

Currently, there is no qualitative information about the impact of COVID-19 restrictions on gender biases in Irish academic institutions. This project aims to analyse the effect of the pandemic on men and women researchers of different career stages. Results will be used to generate a report and will be published in a peer-reviewed journal.

**What is the participant required to do?**

The participant is required to participate in a Zoom interview. The participant will be answering a series of questions related to gender biases and their working conditions before and during the COVID-19 restrictions. Interviews will be recorded, transcribed and anonymised. The data will then be codified according to emergent themes. As a token of appreciation, participants will receive a €25 one4all voucher after the interview. In identifying explanations about gender biases, no potential risks are anticipated. Findings may inform policy development at [UNIVERSITY], which might benefit the participants and the wider [UNIVERSITY] community. If the participant wishes to find out about research results, he/she can indicate that in the informed consent form.

The participant voluntarily takes part in this research project, and can withdraw at any time without providing any reason and can ask for their data to be destroyed.

**Personal Data – GDPR Compliance and Data protection and confidentiality**

The Data Controller is Mohammad Hosseini

The anonymity of participants will be protected at all times and no information will be shared with third-parties. Participants’ gender is the only personal data processed for the research. Recording will be encrypted, password and stored on a secure [UNIVERSITY] Google Drive folder. Transcripts will have all personal details and any other identifications (e.g., institutions’/schools’/persons’ names) removed before being stored on a secure [UNIVERSITY] Google Drive folder. You have the right to lodge a complaint with the Irish Data Protection Commission (<https://www.dataprotection.ie/>) and access your own personal data. Personal contact details will only be used on informed consent forms to allow the research team to contact participants and will be kept in a secure [UNIVERSITY] Google Drive folder. All relevant data will be retained until March 2023 and shall be destroyed by Mohammad Hosseini after this period.

Data made publicly available for conference and journal publications in the field of science and gender studies will be fully anonymised in accordance with the Irish Data Protection Act. Any participant who chooses to withdraw from the study will see all their data immediately destroyed.

Confidentiality of provided information is guaranteed by researchers and data will be protected within the limitations of the law. For further information on the data protection, participants can contact the [UNIVERSITY] Data Protection Officer – [NAME; EMAIL, PHONE NUMBER]

*If participants have concerns about this study and wish to contact the investigator or an independent person, please contact:*

**Mohammad Hosseini, [AFFILIATION] [ADDRESS] [EMAIL]**

**The Secretary, [UNIVERSITY] Research Ethics Committee, [DEPARTMENT], [UNIVERSITY], [ADDRESS, PHONE NUMBER, EMAIL].**

# Informed Consent Form

Research study title: the impact of COVID-19 restrictions on gender bias in academia

**What is this research about?**

In the proposed project, we intend to interview researchers affiliated with the Faculty of [FACULTY] at [UNIVERSITY] to explore *the impact of COVID-19 restrictions on gender bias*. In this research, gender bias is understood as gender-based inclinations or prejudices that affect researchers’ personal and professional opportunities.

**What does the research require me to do?**

I volunteer to participate in a research project conducted by the investigators, Mohammad Hosseini and Alicia Castillo Villanueva. I will be answering a series of questions related to gender disparities and my work environment during COVID-19 restrictions. I will be interviewed via Zoom for no more than 45 minutes and will receive a €25 one4all voucher afterwards.

**Please complete the following (Circle Yes or NO for each question)**

I have read the Plain Language Statement (or had it read to me)?

Yes/No

I understand the information provided?

Yes/No

I have had an opportunity to ask questions and discuss this study?

Yes/No

I have received satisfactory answers to all my questions?

Yes/No

I understand that my participation in this project is voluntary and that I receive a gift voucher after the interview as a token of appreciation for my time. This, however, will not affect my responses or right to withdraw from the research study at any point. If I decline or withdraw from the study the data will be immediately destroyed.

Yes/No

I understand that the interview is thought-provoking and could get personal. If, however, I feel uncomfortable in any way during the interview session, I have the right to decline to answer any question or to end the interview, and ask the interviewer to delete the tape.

Yes/No

I understand that notes might be written during the interview. Audiotape and subsequently transcripts will be made. If I don’t want to be taped, I understand that only notes will be taken.

Yes/No

I understand that the researchers will not identify me by my name in any reports using information obtained from the interview and that my confidentiality as a participant will remain secure. The use of records and data will be subject to standard data protection policies which protect the anonymity of participants. I understand that the confidentiality of information is subject to legal limitations by which [UNIVERSITY] complies with.

Yes/No

I would like to receive information about the research and its results once the study is finished. I understand that I will need to provide my email that will be securely stored.

Yes/No

I have read and understood the information in this form. The researchers have answered my questions and concerns and I have a copy of this consent form. Therefore, I consent to take part in this research project.

Yes/No

Participant Signature: ________________________________________________________

Name:

Date:

# Questionnaire

Short introduction of the interviewers. Our aim today is to explore the impact of COVID-19 restrictions on gender bias in academia.

1. Before the COVID-19 Restrictions:

- Were you aware of any gender inequalities in the society and academia?
- Do you think there were any gender differences in your working environment? [If yes, what do you think was the most salient issue?]
- Did you ever feel that a specific task is delegated to you because of your gender?
- Did you ever feel that your opinion is dismissed or that you are being treated as less professional compared to colleagues of other gender identities?
- Did you ever feel that your colleagues with other gender identities have better chances in academia? (e.g., for personal development, collaboration, publication, career progression and networking)
- How often did you work remotely before the pandemic?
- [In case of having children] were your childcare responsibilities equally shared between yourself and your partner?

2. During the COVID-19 Restrictions:

- Can you tell us about your academic experiences in the last while and the way these were affected by COVID-19 restrictions?
- Have any gender issues been exacerbated/improved due to COVID-19 restrictions?
- [If not single] did you receive enough support from your partner?
- What aspect of your personal life has been affected the most by COVID-19 restrictions?
- [If not answered by the previous question] What has been the most positive/negative effect of COVID-19 restrictions on your work-life balance?
- Do you have a reasonable working space at home? (e.g., separate room?)
- What aspect of your professional life has been affected the most by the COVID-19 restrictions (e.g., teaching, supervision, collaboration, research, publication, grant submission) and why?
- Do you think that negative impacts of COVID-19 restrictions equally impacted colleagues with other gender identities?
- Have you received enough support from your male/female colleagues during COVID-19 restrictions?
- How did you support your man/woman colleagues during COVID-19 restrictions?
- How did your direct supervisor/manager support you during COVID-19 restrictions?
- What kind of support did you receive from your head of school, faculty and/or management?
- Have you heard stories from friends based in other institutions/countries with better/worse experiences?

3. After the COVID-19 Restrictions

- What policies should be adopted by the University to improve gender equality upon returning to normality?
- What policies should be adopted by the University to prevent challenges you faced during the COVID-19 Restrictions?
- What decisions should be made at your home or in your personal life to prevent challenges you faced during the COVID-19 Restrictions?
- 5. Do you have any other issues/points in relation to gender bias in your workspace that was not mentioned in this interview?

# COREQ Checklist

In this section, we report the used methodology using the checklist suggested by Consolidated criteria for reporting qualitative research (COREQ).^[26]^

1. Interviewer/facilitator: Mohammad Hosseini (M.H.) and Alicia Castillo Villanueva (A.C.V.) conducted all the interviews.

2. Credentials: M.H. has a PhD in Research Ethics and Integrity and A.C.V. has a PhD in Gender Studies.

3. Occupation: M.H. is a Postdoctoral Researcher in Ethics and A.C.V is an Assistant Professor in Spanish Cultural Studies, Gender and Sexuality.

4. Gender: M.H. identifies as a man and A.C.V. identifies as a woman.

5. Experience and training: M.H. and A.C.V. are trained researchers with previous interview and qualitative analysis experience.

6. Relationship established: A.C.V. knew of two interviewees but they had not directly worked with them. M.H. knew none of the interviewees.

7. Participant knowledge of the interviewer: Two interviewees knew of A.C.V. before the interview and none knew M.H.

8. Interviewer characteristics: M.H. and A.C.V. introduced themselves in the beginning of each interview and explained study’s goals and objectives.

9. Methodological orientation and theory: Inductive approach as suggested by Thomas, A General Inductive Approach for Analysing Qualitative Evaluation Data (2006).

10. Sampling: purposive.

11. Method of approach: An email invitation to participate in the study was sent to all Faculty staff by the Faculty’s Secretary office on 10 May 2021, and was followed by a reminder on 17 May 2021.

12. Sample size: Nine women and six men were interviewed.

13. Non-participation: N/A.

14. Setting of data collection: Zoom software.

15. Presence of non-participants: N/A.

16. Description of sample: Men and women employees affiliated with a STEM Faculty in an Irish University.

17. Interview guide: The questionnaire was designed by M.H. and A.C.V. and revised after two pilot interviews.

18. Repeat interviews: N/A.

19. Audio/visual recording: Recordings were stored on the cloud on A.C.V.’s password protected Zoom account.

20. Field notes: N/A.

21. Duration: Interviews varied in length from 39:02 to 1:31:14 minutes and lasted an average of 50 minutes and 33 seconds.

22. Data saturation: Saturation was not discussed but given the recruitment limitations, the sample size was deemed reasonable.

23. Transcripts returned: No.

24. Number of data coders: Two, including both authors.

25. Description of the coding tree: The first version of the codebook was developed after analysing three randomly selected interviews. From this analysis 12 codes emerged, most of which were either renamed or merged with other codes. In analysing interviews, the 12 initial codes were revised several times. Ultimately, the authors agreed on subsuming 11 codes under three categories:

1. Gender biases before the COVID-19 restrictions.

- Institutional Gender Disparities: Instances of gender disparity/equality at the University.
- Discrimination: Instances where researchers are subject to unfair treatment by colleagues.
- Implicit bias and stereotypes: Instances where members of a gender group are positively/negatively affected because of implicit biases and stereotypes.
- Gendered Roles: Instances where researchers conduct specific tasks because of their gender.

2. Gender biases during the COVID-19 Restrictions.

- Working from Home: Instances of gender disparity/equality at home
- Parenting: Instances where references to parenthood are mentioned
- Collegial Support: Instances where researchers were/were not supported by colleagues.
- Support from the University. Instances where researchers did/did not receive support from the University.
- Negative Impacts of Restrictions on Existing Gender Biases: Instances where gender biases were exacerbated because of the COVID-19 restrictions.
- Positive Impacts of Restrictions on Existing Gender Biases: Instances where gender biases were improved because of the COVID-19 restrictions.

3. Post COVID-19 Restrictions

- Suggestions to improve gender biases.

26. Derivation of themes: Codes were identified from the data and were not predefined.

27. Software: N/A.

28. Participant checking: N/A.

29. Quotations presented: Where relevant, direct quotations are used.

30. Data and findings consistent: We sought feedback from two external reviewers (Ms. Gretchen Neidhardt and Ms. Shiva Sharifzad).

31. Clarity of major themes: we presented nine more commonly identified codes.

32. Clarity of minor themes: N/A

# Results of the Likert Type Questionnaire

At the end of each interview, the interviewer read the following sentences and asked the interviewee to react using five Likert type responses (i.e., strongly agree, agree to some extent, neither agree nor disagree, disagree to some extent, strongly disagree). The summary of interviewees’ responses is presented in Table 1.

Table 1. Interviewees’ responses to the Likert type questionnaire. Each cell reports the corresponding number of respondents. SA—strongly agree, A—agree to some extent, N—neutral (neither agree nor disagree), D—disagree to some extent, SD—strongly disagree.

| **Statement** | SA | A | N | D | SD |
| --- | --- | --- | --- | --- | --- |
| During the COVID-19 restrictions, I felt supported by my spouse/partner.^*^ | 7 | 3 | 0 | 0 | 0 |
| During the COVID-19 restrictions, I felt supported by my supervisor/manager. | 4 | 6 | 3 | 1 | 1 |
| During the COVID-19 restrictions, I felt supported by man colleagues. | 4 | 8 | 0 | 1 | 2 |
| During the COVID-19 restrictions, I felt supported by women colleagues.^**^ | 4 | 9 | 0 | 0 | 1 |
| During the COVID-19 restrictions, I felt supported by my head of school. | 5 | 5 | 2 | 0 | 3 |
| During the COVID-19 restrictions, I felt supported by non-academic staff. | 9 | 6 | 0 | 0 | 0 |
| Since a large proportion of communication during the COVID-19 restrictions happens through email, I feel that the chances of bullying and harassment was reduced. | 0 | 2 | 5 | 6 | 2 |
| Restrictions had a negative effect on chances to publish my work. | 7 | 3 | 2 | 2 | 1 |
| Restrictions had a negative effect on chances to access information. | 6 | 3 | 3 | 2 | 1 |
| Restrictions had a negative effect on chances for international collaborations. | 0 | 6 | 2 | 4 | 3 |
| Restrictions had a negative effect on chances for grant submissions. | 3 | 8 | 0 | 3 | 1 |
| Restrictions had a negative effect on chances for career progression. | 4 | 4 | 2 | 3 | 2 |

* This question was not asked of our five single interviewees.

** One interviewee had no women colleagues.
