# Supplementary material for: A qualitative analysis of the impact of COVID-19 restrictions on gender biases in an Irish University
Source: PLoS One. 2023 Sep 27;18(9):e0288467. doi: 10.1371/journal.pone.0288467 (PMC10529590; doi:10.1371/journal.pone.0288467)
Supplement: Attachment — Submitted filename: PONE-D-22-26961R1 Review.docx [file pone.0288467.s002.docx]

PONE-D-22-26961R1	Review

The article presents a qualitative small-scale study on perceptions of the impact of COVID-restrictions on gender bias in an Irish University STEM Faculty based on semi-structured interviews with 15 academic women and men. The authors conclude that gender biases experienced before COVID-19 restrictions were “different from biases during restrictions” (abstract), and that women researchers with caring responsibilities have been mostly affected by the COVID-restrictions. They also present future visions on how to reduce gender disparities, including “promoting horizontal organisational structure” (abstract), adjusting policies and work arrangements to support vulnerable groups, and introducing hybrid working models.

The main weakness of the article is that it is rather thinly relating the findings and discussion of results and future visions to extant and extensive literature on gender inequalities in academia, including much recent published research focusing on Irish academia (e.g. Drew, E., & Canavan, S. (2020). *The Gender-sensitive University: A Contradiction in Terms?*. Taylor & Francis). Furthermore, thinking of international readers who may not know the Irish context, the authors fail to characterize the Irish national background: the pro-active gender equality approach of Irish higher education with very recent national reviews (2022), the more so relevant given that Ireland belongs to the EU countries which during the recent decade have made notable advances in national policy in this area (see, e.g. EU She Figures 2022). The article needs major revision in these respects, including specifying better when to use which gender concepts (gender equality/equity, gender balance, gender disparities, gender bias, gendered roles). Gender equality/equity is more than gender balance, for example.

2. Method. The authors do not spell out in the beginning of the methodology subchapter what method is used for data collection but speak about “questionnaire” which is a tool, not a method and could refer to many things. It comes clear only later when reading ahead and consulting additional material, that the data collection method is thematic interview.

The sampling was purposive, and the invitation for interview was sent to all academic and support staff within the chosen Faculty. The authors received only 15 responses and considered it satisfactory, but they do not tell how many invitations were sent in total, and how many women/men were in the staff overall? These details should be explained to be able to assess the consequences for the sampling – was it a biased sample and into what direction?

The authors present necessary technical details on the interviews in the supplementary document. Some of that data could be lifted to the article text to make the methodology subchapter richer. I was wondering how the authors introduced themselves in the interviews – this is rather relevant in this kind of study where academics study academics and does influence trust and informants’ willingness to disclose. What I also missed was some characterisation on the nature and flow of the interviews concerning the ambience of the interviews: hesitations, short answers, emotions?

The socio-economic data table 1: how many identified to belong into LGTBQI+ at least two according to the results part – would be relevant concerning comments on these issues in reporting. Why married a category but not cohabiting? May be more relevant to know how many were single than formally married.

5. The article is generally well and clearly written but the authors should check for typos.

Additional comments:

The meaning of Athena Swan accreditation in the Irish HEI context needs to be explained to international readers, maybe in a footnote.

Figure 1. It is not clear what is the relevance of this figure beyond mere illustrating the topic.

Claim that LGTBQI issues represented “by one specific subgroup” – could the authors tell which? P.10

Gendered roles subheading (p.12) – role terminology may not be the best option here: there is much extant literature on gendered division of labour in academia, and for some aspects of results the concept “academic housework” that is gendered would be more relevant than using the role concept.

p.19. Negative impacts on gender biases by Covid restrictions – no gendered implications? I read the quotes somewhat differently - women reporting more difficulties getting one’s voice heard, and one man commenting more general level suggests gendered patterns – small sample though.

In Discussion: The statement “While the transition to online environments

removed the possibility of being harassed and made communication more measured

and cautious, it amplified a top-down approach.“ This is not a correct claim. According to much recent research, online environments are far from being free from harassment.
